# Oral health problems facing refugees in Europe: a scoping review

**DOI:** 10.1186/s12889-021-11272-z

**Published:** 2021-06-24

**Authors:** Eiad Zinah, Heba M. Al-Ibrahim

**Affiliations:** 1 Dental Public Health Department, University College London, London, UK; 2 Department of Orthodontics, University of Damascus Dental School, Damascus, Syria

**Keywords:** Oral health, Dental caries, Periodontal disease, Refugees, Asylum seekers, Europe

## Abstract

**Introduction:**

Europe has been experiencing a flow of refugees and asylum seekers driven by conflicts or poverty. Their oral health is often neglected despite its clear impact on quality of life.

**Objective:**

To explore the status of oral health among refugees and asylum seekers groups by examining the available literature and to determine which evidence exists regarding the problems they face in terms of oral health.

**Methods:**

The current paper followed PRISMA guidelines. A scoping review methodology was followed to retrieve 2911 records from five databases and grey literature. Twelve articles met the following inclusion criteria: experimental research concentrated on the oral and dental health of refugees and/or asylum seekers between 1995 and 2020 in English. Analysis was both descriptive and thematic, whilst a critical appraisal was applied using the Critical Appraisal Skills Program (CASP).

**Results:**

Seven studies (58,3%) were quantitative, while five studies (41,6%) were qualitative. In general, the quality of most of the studies (83.3%) was good. Limited access to oral health care services was shown with a higher prevalence of oral diseases compared to the native populations of the host countries. Approaches to improve oral health have been implemented in some studies and have shown positive outcomes.

**Conclusions:**

Oral health care strategies should consider the oral health problems facing refugees in Europe, and oral health promotion campaigns are essential to give adequate guidance on how to access oral health care in the host countries.

**Supplementary Information:**

The online version contains supplementary material available at 10.1186/s12889-021-11272-z.

## Background

Oral diseases are considered one of the most predominant diseases worldwide, affecting the population’s quality of life, health complications, as well as many dangerous loads on the economy [1]. The most common and significant oral diseases are teeth caries, periodontal diseases and oral cavity lesions [1]. Oral diseases affected 3.9 billion people in 2010 with the most prevalent diseases being untreated decays with a global predominance of 35% for all ages [2]. The situation worldwide is terrible, where one in three persons has untreated decay in one or more of the permanent teeth [3]. About 2.4 billion people were estimated to have decays of permanent teeth globally, and 486 million children have deciduous teeth caries [4]. Treating dental problems has prohibitive costs, for example, according to the Medical Expenditure Panel Survey announced in 2006, approximately 19% of children had dental expenses of $729 million [5].

The number of refugees and asylum seekers’ continues to increase around the world [6]. Approximately 19.5 million refugees and 1.8 million asylum seekers were existent worldwide by the end of 2014 [6]. But research to find out about their oral health needs and inform policymakers concerning access to oral health care is still limited. Refugees and asylum seekers’ oral health is an important issue, but it has not become a priority globally [7–9]. Access to oral health care has been one of the problems faced by refugees in Europe, and not much is known about the overall prevalence of oral diseases and their causes for this part of the population [10–12]. Oral health referred to self and professional evaluation of the oral health situation, personal attitudes to oral care and oral hygiene behaviors, and access to care, including barriers preventing refugees and asylum seekers from getting professional oral health care services [13–15]. The limited numbers of available studies have shown that oral health was poor among refugees and asylum seekers [16]. Factors such as inadequate standard healthcare systems in some countries, difficult journeys that this group of people take to new countries, and individual oral health behaviors and practices cause many oral health problems and poor dental health outcomes, which might lead to negative effects on general health and quality of life, and could rise the risk of chronic diseases [17]. For example, pain from a diseased tooth can restrict eating, which compromises nutrition, and periodontal disease is associated with diabetes and cardiovascular disease [16]. However, acculturation is a dynamic way in which persons face psychological and cultural modifications [18]. This acculturation has many components that help people with integration, such as learning a new language and adapting to standards and traditions [18].

The healthy migrant effect was studied by many researchers who have found that migrants (including refugees) often receive more health services and have more approving health status than the population in the original country [19]. However, that effect heads for diminishing over time due to different reasons, such as financial hurdles [20].

The scoping review conducted by Keboa et al. in 2016 [21] evaluated the oral health of refugees worldwide without considering the proportions of the refugee and their distribution according to the regions. The dental services provided differ from one continent to another and from one country to another, which may be reflected in the accuracy of the results. Also, this review has been published for more than 4 years; therefore, the recently published literature has not been evaluated.

### Purpose

The aim of this work was to present a scoping review of the studies that have been done by researchers in Europe in recent years and to map available literature on the oral and dental health of asylum seekers and/or refugees in Europe. The objectives of this review were: (i) to appraise the chosen studies critically; (ii) to define and characterize the oral diseases predominance among asylum seekers and refugees; and (iii) to outline services and strategies to promote oral and dental health of this group.

The main question of this review was: What are the important issues related to oral and dental health and the oral care services available to refugees and asylum seekers in Europe? Oral health referred to self and professional evaluation of the oral health situation, personal attitudes to oral care and oral hygiene behaviors.

## Method

This scoping review was carried out between June and September 2018 and updated in January 2021. The adopted methodological framework in the current scoping review was the revised Arksey and O’Malley framework, which has five major steps, in addition to Levac et al., steps [22, 23]. The consultation exercise, which is the final optional step in Arksey and O’Malley’s framework, was not done in this review. This framework provides a foundation for scoping study methodology. The approach adopted by Kebo et al. who included a quality assessment was also followed [21].

### Identifying relevant studies

With the assistance of a UCL university-based librarian who provided assistance regarding the methods of searching, a comprehensive search of peer-reviewed and grey literature to find relevant publications was undertaken. The search strategy in Medline Ovid was applied using MeSH terms and keywords, as shown in Additional file 1, and the same search was conducted in PubMed, Embase, Global Health, and Scopus. Websites of international and European organizations working with refugees, such as Health-Point Foundation (HPF), a volunteer-led charity whose mission is to provide dental treatment for refugees, were also included to perform a grey literature search. Finally, Google Scholar was used to ensure full coverage of the relevant publications or articles.

### Study screening and selection

**Fig. 1:**
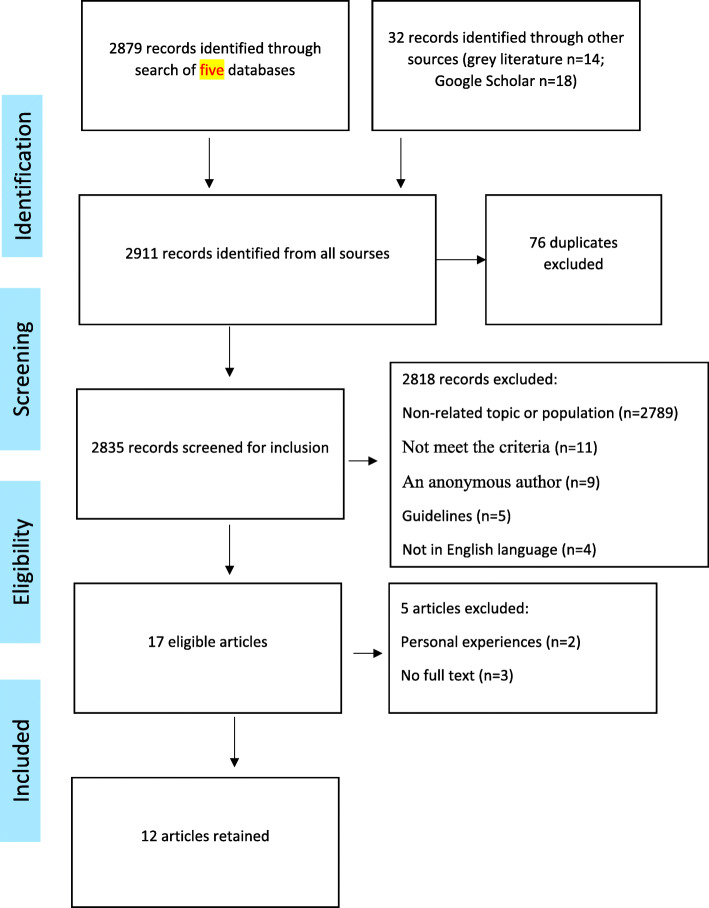
PRISMA flowchart of study selection

The screening and selection procedure shown in Fig. 1 was applied using the preferred Meta-analysis (PRISMA) flowchart [24]. A total of 2911 references were acquired then duplicates (*n* = 76) were excluded using the EndNote reference manager. The remaining 2835 references were screened by two reviewers, using the following inclusion criteria: (i) all study designs of articles were accepted, (ii) the studied participants had to be refugees and/or asylum seekers without restrictions on the age group or gender, (iii) the host country has to be a European country, (iv) articles had to study one of the oral and dental health aspects, and (v) articles had to be published in English between 1995 and 2020. A refugee was described as an individual who has escaped his/her country of origin to ask for security in another country and his/her refugee status was admitted [25]. On the other hand, an asylum seeker was described as a person who has applied for refugee status, but the asylum application has not been accepted yet [25].

Seventeen studies were finally retained. The full text of only 14 could be found. Further two studies were excluded (personal experiences) after reviewing. In conclusion, 12 articles met the inclusion criteria of experimental research focused on refugees’ and/or asylum seekers’ oral health and then went towards the critical appraisal.

In the event of any discrepancies during the screening, full-review process, collating, summarizing, and reporting the results, the opinion of an experienced third person was taken.

### Charting the data

The following data was charted from the 12 remaining studies where possible: i) Bibliographic information (first author, year of publication, title and journal); ii) category of the source and article; iii) The theories and frameworks used; iv) Objectives and aims, type and study design, country and duration of the study, sampling, target groups, tools used for data collection, analysis method, results and recommendation.

### Collating, summarizing, and reporting the results

The three steps according to Levac et al. [23] to generate results were followed: (i) information of each article was included in one table; (ii) information was obtained by the two reviewers; then the descriptive analysis was done; (iii) comparable data parts were combined and analyzed thematically [26].

### Quality appraisal

A Critical Appraisal was performed using the Skills Programme (CASP) recommended by Oxford University [27]. This programme consisted of 12 questions to evaluate the quality of quantitative studies and 10 for qualitative studies. Good vs satisfactory was used as a rating system to evaluate the studies.

## Results

### Descriptive analysis

**Fig. 2:**
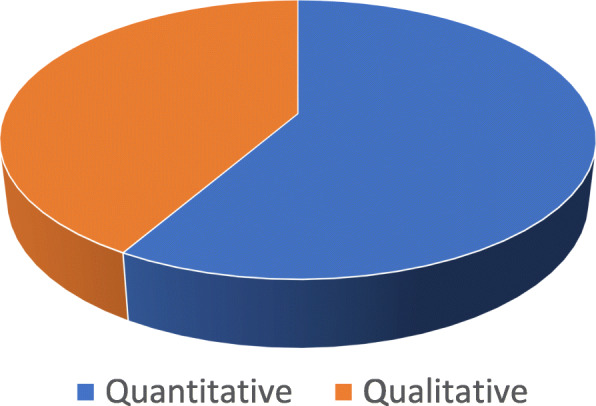
Distribution of study designs

**Fig. 3:**
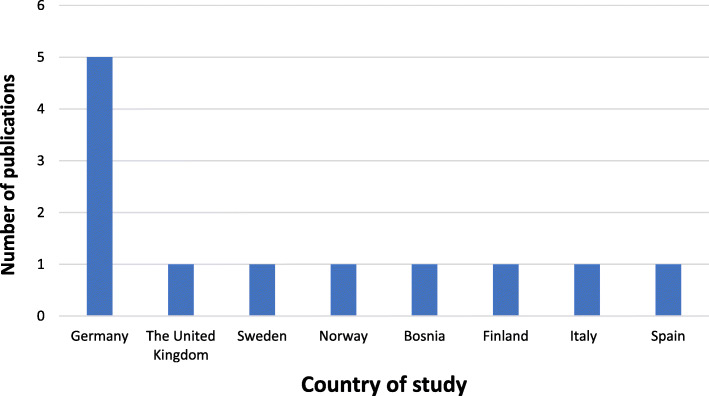
Number of studies per country

**Fig. 4:**
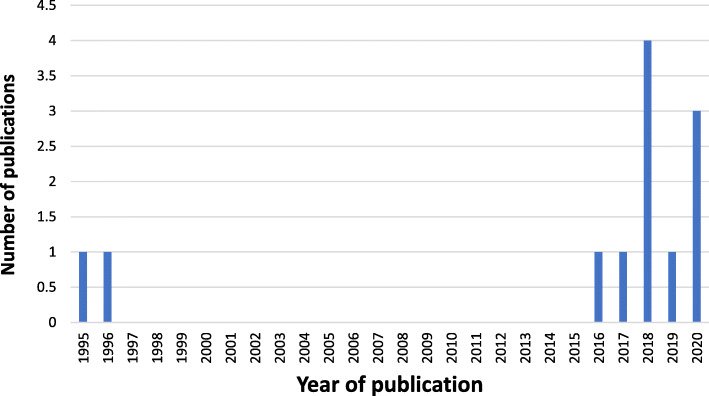
Number of publications per year

After looking at the 12 remaining studies for the review, it was found that 7 (58,3%) of the articles were quantitative, and 5 (41,6%) articles were qualitative (Fig. 2). Eleven studies were from Western and Northern European countries, and One study was from the UK: 5 studies were in Germany, 1 in the United Kingdom, 1 in Sweden, and one in Norway, Bosnia, Finland, Italy and Spain (Fig. 3). Ten studies were published between 2016 and 2020, while two were published earlier (Fig. 4). In general, the quality of the studies (83.3%) were good. National and international journals that focus on different health issues published eight articles, and the other four articles were published in dental specific journals. The studies had participants originating from countries in the Middle East, Africa, Asia and Latin America. Two studies focused on oral health in children refugees [10, 28], four assessed the oral health situation of refugees and the prevalence of caries and other oral diseases and the treatment needs [11, 28–30]. The gender was balanced in the studies. Three studies focused on access to dental care and the use of dental services among refugees [28, 31, 32] and one study compared oral health behaviors between migrants and non-migrants [9]. In addition, another study was about the cost of dental care for refugees [15]. One of the studies presented the oral health challenges for this group of people [8]. Finally, one study evaluated the fear and anxiety for dental health among displaced people in Bosnia and Herzegovina [33]. Studies characteristics are summed in Table 1. The quality appraisal of the quantitative studies included in the assessment showed that some were cross-sectional [9, 11, 15], one cohort study [10], one retrospective observational study [28], one retrospective hospital-based study [28], and one pilot study [32].

**Table 1:** Characteristics of included articles (*n* = 12)

Study	Location	Study Design	Study Purpose	Participants			
				Age	Gender	N	Nationality
Fennell-wells (2020) [12]	UK	A review of the asylum process in the UK, the oral health status of child asylum seekers and the challenges in accessing dental services has been conducted.	To provide a summary of the oral health status of child refugees and asylum seekers, and to describe their access to health services	N/A	N/A	N/A	N/A
Freiberg (2020) [28]	Halle (Saale), Germany	Retrospective observational study	To quantify the utilization of dental health services, the complaints leading to visits at the dental clinic, the diagnoses made by the dentist, and the treatments resulting from it.	20–34 Years old	Males and females	4107	Syria, Afghanistan, Iran, Somalia, Guinea-Bissau, Russian Federation, Eritrea, India, Kosovo, Benin, others
Al-ani (2020) [9]	Four federal states in Germany	Quantitative cross-sectional study	To assess oral health, caries prevalence, and subsequent complications among recently arrived refugees, and to compare these findings with the resident population.	3–75+ years old	Males and females	544	Syria, Afghanistan, and Iraq Egypt, Mauritania, Lebanon, Palestine, Morocco Kosovo, Albania, Ukraine, Armenia, Serbia, Cheek Republic, Georgia, and Macedonia Iran, Pakistan, Thailand, Azerbaijan, Tajikistan, and Russia Eretria, Ghana, Nigeria, Ethiopia, and So
Høyvik (2019) [8]	Oslo/Norway	Twelve qualitative interviews as well as participant observation	To explore experiences of irregular migrants related to their oral health and their access to dental care	29–50	Males and females	10–20	Not mentioned
Solyman (2018) [11]	Germany	Quantitative study: Cross-sectional (and structured interview conducted)	To determine the status of oral health among newly arrived refugees and to explore their knowledge, attitude and practices on oral hygiene	18–60 years old	Males and females	386	Syria and Iraq
Zukanović (2018) [33]	Canton Tuzla, B&H	Qualitative study; Narrative Interviews	To evaluate the DFA presence and the most common reasons for dental fear and anxiety in displaced persons	between 35 and 44 years old	males and females	310	Several cities of Canton Tuzla Bosnia and Herzegovina.
Goetz (2018) [15]	Schleswig-Holstein, Germany	Pilot study with a cross-sectional	To evaluate the oral health of refugees and to estimate the costs of oral care.	a mean age of 28 years old	Males and females	102	Afghanistan, Iraq, Syria, Eritrea, Yemen, Armenia, Somalia, Iran, Chechnya
Riatto (2018) [10]	Melilla, Spain	Quantitative study/Correlation questionnaire	To determine the oral health status of Syrian immigrant children refugee at the Center for Temporary Stay	5–13 years old	males and females	156	Syria
Furnadzhieva (2017) [30]	Baden-Württemberg, Federal Republic of Germany.	Qualitative study	To study the experience of dental practitioners in Germany in treating refugees at outpatient medical facilities	5 to 75 Years old	N/A	100	N/A
Mattila (2016) [32]	Finland	Pilot study	To investigate self-reported oral health, oral health habits, dental fear and use of dental health care services among asylum seekers and immigrants	17 to 53 Years old	Males and females	38	15 different countries
Angellilo (1996) [29]	Catanzaro and Crotone. /Italy	Quantitative study	To assess the caries prevalence, oral hygiene status, periodontal health and the treatment needs in immigrants and refugees	18–44+ years old	Males and females	252	Senegalese, Yugoslavs, Moroccans
Zimmerman (1995) [31]	Sweden	Quantitative-Cross Sectional study	To describe and analyze consumption of dental care in different refugee groups	23–34 years old	Males and females	2489	different countries

*N* number of participants, *N/A* not applicable

### Quality appraisal

The qualitative and quantitative studies meet most of the acceptable number of CASP criteria, objective or study purpose, age of participants and study population, as well as, study location was clearly mentioned. Furthermore, five of the quantitative studies reported the statistical significance of the results and tested for *p*-value [9, 11, 15, 29, 31] and the same number of these quantitative studies calculated a confidence interval around the results [10, 11, 15, 28, 29]. Three qualitative studies used qualitative description [12, 32, 33], while two studies used interviews and a phenomena graphic approach to collect and analysis the data [30] (Tables 2, 3).

**Table 2:** Quality appraisal of the qualitative papers

First author (year)	CASP criteria satisfied	Unclear criteria	CASP criteria Unmet	Proportion of satisfied criteria (n%)	Assessment	Main criteria not achieved
Fennell-wells (2020) [12]	7	2	0	7/9 (77.7%)	Good	N/A
Høyvik (2019) [8]	7	3	2	7/12 (58.3%)	Good	No Confidence Interval calculated
Furnadzhieva (2017) [30]	8	2	0	8/10 (80%)	Good	Relationship between researcher and participants not mentioned
Zukanović (2018) [33]	5	3	2	5/10 (50%)	Satisfactory	Validity of questionnaire not mentioned
Mattila (2016) [32]	5	4	1	5/10 (50%)	Satisfactory	Relationship between researcher and participants not mentioned

**Table 3:** Quality appraisal of the quantitative papers

First author (year)	CASP criteria satisfied	Unclear criteria	CASP criteria Unmet	Proportion of satisfied criteria (n%)	Assessment	Main criteria not achieved
Riatto (2018) [10]	8	4	2	8/12 (66.6%)	Good	No Confidence Interval calculated
Zimmerman (1995) [31]	10	1	1	10/12 (83.3%)	Good	N/A
Freiberg (2020) [28]	7	3	2	7/12 (58.3%)	Good	N/A
Solyman (2018) [11]	8	2	2	8/12 (66.6%)	Good	Relationship between researcher and participants not mentioned
Angellilo (1996) [29]	8	2	2	8/12 (66.7%)	Good	No confidence intervals Calculated
Al-ani (2020) [9]	10	1	1	10/12 (83,3%)	Good	N/A
Goetz (2018) [15]	9	2	1	9/12 (75%)	Good	N/A

### Thematic analysis

#### Oral health understanding, knowledge, behaviors, practices and beliefs

Refugees in Europe may face many serious personal barriers in accessing dental care, including low income, fear or anxiety regarding treatment, language barriers, educational and cultural barriers such as differences in understanding oral health concepts, different beliefs about dental care, and lack of knowledge about health care services in a foreign country [34]. In refugee camps in most host countries in Europe, there is limited access to oral health care services due mainly to a shortage of dental professionals often resulting in oral health care service being limited to just tooth extraction [11].

Previous oral health care experiences of refugees in their countries of origin and beliefs they may hold can influence their oral hygiene practices. In addition, the process of migration and integrating into a new culture can have a disruptive impact on the use of dental services [8]. Two articles focused on understanding the refugee experience of pre-school children in terms of early oral health and experiences accessing dental services [10, 12]. These two articles mainly focused on early childhood caries and were published in the last 2 years. The main factors that were found to be significant were the influence of the parents’ previous experience, their beliefs and understanding of deciduous teeth and lack of knowledge in the importance of early dental care and oral health. Prevailing unhealthy habits of eating, such as consuming too much sugar, fizzy drinks, and snacks, could be a risk for tooth decay and could contribute to periodontal disease and then poor oral health. Furthermore, poverty was found to be significant in contributing to poor oral health too. Pressing challenges of resettlement give priorities to other things and can result in oral health being overlooked, and this was mentioned almost in all studies. Additionally, difficulties in accessing dental services and language barrier were a significant factor preventing refugees from getting dental treatment [10, 11].

Furnadzhieva et al. and Høyvik et al. concluded that parents were reluctant to adopt a preventive approach to oral health and only took their children to the dentist when they were in pain [8, 30]. Zimmermann et al. demonstrated that many adults estimated their oral hygiene to be much better [31].

In one example from a study in Germany, participants assessed their oral health to be of an acceptable level, yet the clinical examinations showed 80% with periodontal disease and untreated decay [9]. Some studies found that beliefs and culture could negatively impact refugee’s oral health [29]. Solyman et al. found there was sound knowledge of oral health amongst Syrian and Iraqi refugees but also a gap between this and their oral hygiene practice [11]. In terms of diet, Mattila et al. and Freiberg et al. showed that refugee’s eating and drinking habits have changed after their arrival in Europe and that sugar intake increased, which influences their teeth [28, 32]. Zukanović et al. showed that the level of dental fear and anxiety has been higher in the group of displaced persons due to their bad experiences [33].

#### Oral health problems, disease and treatment needs

Høyvik et al. found that oral health challenges affected refugees’ life quality in Norway: dental pain led to frustration and anger, and missing teeth hindered refugees’ ability to learn a new language [8]. The same study also found that 50% of participants complained that poor oral health had a negative impact on their daily lives at least once per week [8]. The most assessed oral disease covered in the studies was dental caries [9, 10, 28, 29], but periodontal diseases were another common noticeable disease [28, 30], in addition to enamel fluorosis, oral lesions and dental injuries [12]. Assessments for these oral diseases were performed in different places such as dental clinics [30], hospitals [28] and community organizations [15, 29].

By looking at all these studies, the refugee samples had a relatively high rate of oral disease. Although the prevalence of these diseases differed between the studies, the levels of diseases were always higher for refugees compared to levels reported for the wider populations of the host countries. For those reasons, better knowledge and professional assessment of dental treatment were strongly recommended for this population. Even though perceived treatment needs were different between studies, they were described as urgent in all of them. Generally, treatment of dental decay and periodontal disease was the most urgent treatments mentioned in the studies.

#### Implications and strategies to improve oral health

Evidence shows that providing information on oral health and diet in the refugees’ language has led to improvements in oral health and lower sugar consumption [11]. Oral health educational campaigns, including group work, might be useful and could impress refugees on the importance of oral health and preventative dentistry [11, 28]. Furthermore, it states the need for clinicians to explain clearly for refugees how to improve their oral health [28]. More generally, strategies to improve oral health in refugees can be classified into three different categories: (i) service provision; (ii) educational; and (iii) emergency training [35]. In brief, educational strategies should improve refugees’ oral health via health-promotion sessions and printed information. Service provision combines personal oral healthcare instructions with free oral healthcare given by volunteers, perhaps in mobile dental units [36]. Emergency training involves training refugees themselves in providing short-term solutions to urgent problems for themselves or members of their community.

## Discussion

The review aimed to present the important issues related to oral and dental health and the oral care services available to refugees and asylum seekers in Europe.

Refugees suffer from many difficulties related to resettlement in the new community [18]. Acculturation has many components, such as learning a new language and adapting to standards and traditions, which may help people integrate, alleviating the difficulties and pressures refugees face [18].

About 60 % of studies were quantitative, reflecting on the increasing awareness over the last few years of the need for qualitative data to improve the oral health interventions and results [37]. Most of the studies were from Scandinavian and Western European countries, and it was rare to find any research relating to the oral health of refugees from countries in Southern or Eastern Europe. However, most refugees and asylum seekers choose to resettle in Northern and Western European countries [38]. The main challenge was in recruiting participants and difficulty to reach populations; for that reason, researchers selected a convenience sample when they worked with such populations in order to make the sample sizes meet CASP criteria. It is clear, particularly from the fact that sample sizes did not meet CASP criteria, that research about the oral health of refugees is still limited and the oral health needs and problems of this group of the population is still not well known. The research in Europe related to this topic is very little compared to studies about the same issue in Australia, Canada or the USA. Likely, the health authorities in many countries in Europe are not giving priority to work on prevention and treatment of oral disease [39]. Research on the oral health of this population is important to suggest appropriate dental public health actions. Only one study was conducted on the oral health of refugees in camps [10], so this should be an significant matter for future study.

Periodontal disease and dental caries were the most frequent conditions assessed in the studies, but more attention is needed for other oral health conditions and issues. Moreover, none of the studies reviewed assessed traumatic injuries in the face or the mouth region nor examined the incidence of oral lesions such as oral ulcers, nor the impact of poor oral health on the refugees’ wider life.

Zukanović et al. and Alani et al. looked at the experiences of oral health care among refugees in their host countries and found that dental pain experiences and the fear of dental treatment made them less likely to visit dentists in the host country [9, 33]. This reticence was increased by linguistic barriers and being unable to adequately explain what they were feeling. This demonstrates the importance of improving communication between clinicians and refugees using interpreters when necessary and issuing information leaflets in the refugees’ mother-tongue, especially in relation to diet and the oral-health effects of sugar [30].

To understand the oral health perspectives of the refugees and asylum seekers, more studies are needed, whether using quantitative, qualitative or a mix of these two methods. It is important that future studies focus on understanding the specific differences and beliefs in order to provide targeted and effective interventions. Further, we can expect that different countries in Europe have different health care policy situations, which means providing different dental services for refugees and asylum seekers appropriate to different European countries. Even though oral health policy exists in some European countries to facilitate access to oral care for refugees, these policies do not all get regularly reviewed and improved [12].

The results from this scoping review highlight the most common oral health problems faced by refugees in Europe: the limited access to oral health care, the prevalence of dental caries and periodontal disease among this population, the limited use of preventive oral health services, and the high cost of dental treatment which has led refugees sometimes to settle for tooth extraction despite restoration is possible, either because they are not able to pay for the dental treatment, long waiting times to see a dentist or language barriers.

### Study limitations and strengths

Literature available electronically was explored, but relevant studies not archived electronically could have been missed. However, the search was comprehensive and tried to provide a broad picture of the oral health problems among refugees in Europe. The age of research included in the inclusion criteria was not limited to recent studies; however, this reflects the low quantity of research available on this topic. The quality appraisal applied in the current review is considered one of the strength’s factors.

## Conclusions

Oral health disease is still a challenge for refugees and asylum seekers in Europe. The research level in recent years is in increasing, and the number of people who have an interest in this field become more. Interventions and strategies need to be developed to reduce oral health inequities in this population, and the host countries need to design strategies to improve access to oral health care for refugees and asylum seekers significantly. Further studies and research on the oral health problems of refugees and asylum seekers living in Europe and particularly in refugee-hosting centers around Europe is urgently needed.

## Supplementary Information

**Additional file 1:.** Medline Ovid search strategy.

## Data Availability

The datasets used and/or analyzed during the current study are available from the corresponding author on reasonable request. The five databases with their direct links are: Medline Ovid: https://www.ovid.com/product-details.901.html PubMed: https://pubmed.ncbi.nlm.nih.gov Embase: https://www.embase.com/login Global Health: https://www.ebsco.com/products/research-databases/global-health Scopus: https://www.scopus.com/home.uri

## Abbreviation

UCL: University College London
