# Supplementary material for: Oral health problems facing refugees in Europe: a scoping review
Source: BMC Public Health. 2021 Jun 24;21:1207. doi: 10.1186/s12889-021-11272-z (PMC8223339; doi:10.1186/s12889-021-11272-z)
Supplement: Supplementary file 1 — Additional file 1:. Medline Ovid search strategy. [file 12889_2021_11272_MOESM1_ESM.docx]

| **Additional file 1**: Medline Ovid search strategy | |
| --- | --- |
| 1 | exp Oral Health/ |
| 2 | exp Periodontal Diseases/ |
| 3 | exp Tooth Diseases/ |
| 4 | exp Dental Health/ |
| 5 | exp Dental Care/ |
| 6 | or/1-5/ |
| 7 | exp “Emigrants and Immigrants/ |
| 8 | exp Refugees/ |
| 9 | immigrant*.ti,ab,kw./ |
| 10 | refugee*.ti,ab.,kw./ |
| 11 | or/7-10/ |
| 12 | exp Europe/ |
| 13 | 6 and 11 and 12/ |
| 14 | limit 13 to yr="1995 -2020" |
